# A case of prolonged generalized exanthematous pustulosis caused by hydroxychloroquine—Literature review

**DOI:** 10.1002/ccr3.1811

**Published:** 2018-10-26

**Authors:** Fatemeh Mohaghegh, Minoo Jelvan, Parvin Rajabi

**Affiliations:** ^1^ Assistant Professor of Dermatology and Dermatopathology Department of Dermatology Isfahan University of Medical Sciences Isfahan Iran; ^2^ Dermatology Resident Department of Dermatology Isfahan university of Medical Sciences Isfahan Iran; ^3^ Professor Department of Pathology Isfahan University of Medical Sciences Isfahan Iran

**Keywords:** acute generalized exanthematous pustulosis, hydroxychloroquine

## Abstract

Acute generalized exanthematous pustulosis (AGEP) is a self‐limited drug reaction. Hydroxychloroquine (HCQ) is an uncommon cause of AGEP with a prolonged recovery course; Thus, the physicians should take the possibility of this rare but severe event in their minds and try to diagnose correctly and better management.

## 1 INTRODUCTION

Acute generalized exanthematous pustulosis (AGEP) is a rare cutaneous eruption that often induced by drugs and >90% antibiotics (mainly beta lactams) are most frequent triggers.^1^

Acute generalized exanthematous pustulosis is characterized by acute onset of wide spread nonfollicular pinpoint aseptic pustules overlying erythematous skin. This cutaneous eruption often accompanied by fever (38°C <), leukocytosis and spontaneous resolution within < 15 days that typically followed by desquamation. Frequent pathological features include sub/intracorneal pustules contain neutrophils and eosinophils, necrotic keratinocytes, papillary edema, spongiosis, dermal neutrophilic infiltration with eosinophils and generally absence of vasculitis.^3^ No specific treatment is available. Although in more severe and prolonged cases, systemic corticosteroids are usually administered.^4^, ^5^ Although the pathogenesis of AGEP is not yet known, a role of drug‐specific T cell has recently been proposed.^6^, ^7^

Hydroxychloroquine (HCQ) is an antimalarial drug that has been widely used in dermatologic and rheumatologic diseases and is reported as a rare cause of AGEP.

We presented a case of prolonged HCQ‐ induced AGEP that the eruption waxes and wanes and cleared after gradually tapering of corticosteroid within 68 days.

We also reviewed the literature in English from 2008 to 2018 and found nine other cases that developed AGEP secondary to HCQ.^7^, ^8^, ^9^, ^10^, ^11^, ^12^, ^13^

## 2 CASE REPORT

A 44‐year‐old white woman with a 5‐month history of distal joint pain was started on HCQ 200 mg daily. Five days after initiation of HCQ, the patient developed pruritic erythematous patches with pustules on upper chest and upper limbs.

Despite topical steroids, the lesions persisted and deteriorated. She visited her primary rheumatologist after 10 days, and she presented to rheumatology clinic. HCQ was immediately withdrawn after 10 days. She was started on 30 mg prednisolone daily and was visited by the dermatologist. Skin biopsy, stopping of HCQ, and supportive treatment including antihistamines, topical steroids, and intravenous hydration were planned for her.

**Figure 1:**
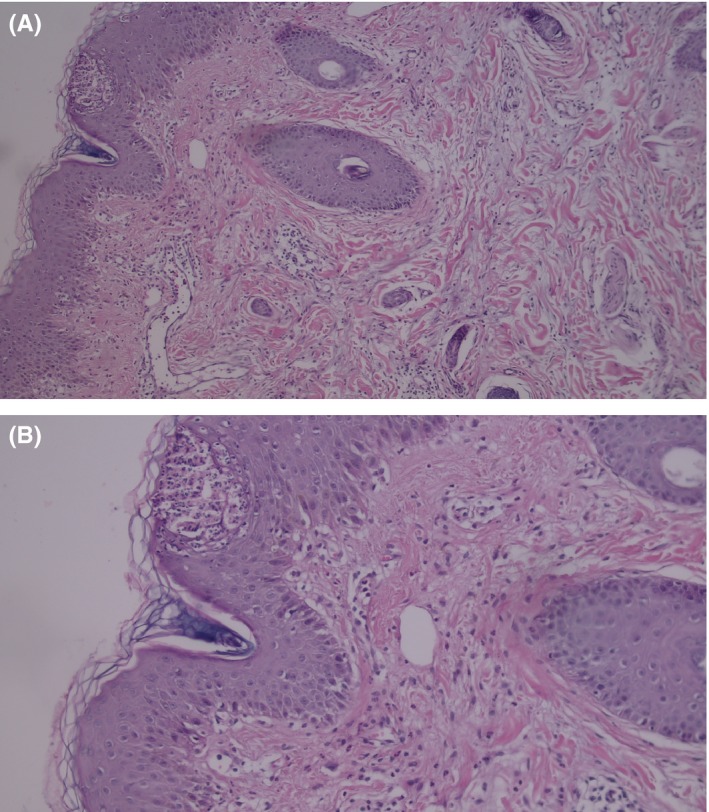
Superficial intraepidermal pustules and perivascular and interstitial mixed infiltrate containing neutrophils, lymphocytes, and some eosinophils in the upper dermis. Hematoxylin and eosin, original magnification : (A) ×40, (B) ×100

Skin biopsy demonstrated nonfollicular Superficial pustules in the epidermis filled with neutrophils, a mixed eosinophilic and neutrophilic perivascular infiltration and absence of psoriasis‐like changes that consisted with AGEP (Figure 1).

After moderately controlled the lesions, the patient was discharged and 30 mg prednisolone was planned to taper gradually by 5‐10 mg weekly, 2 weeks later when the patient was treated on 20 mg prednisolone, once daily she attended the dermatology clinic.

**Figure 2:**
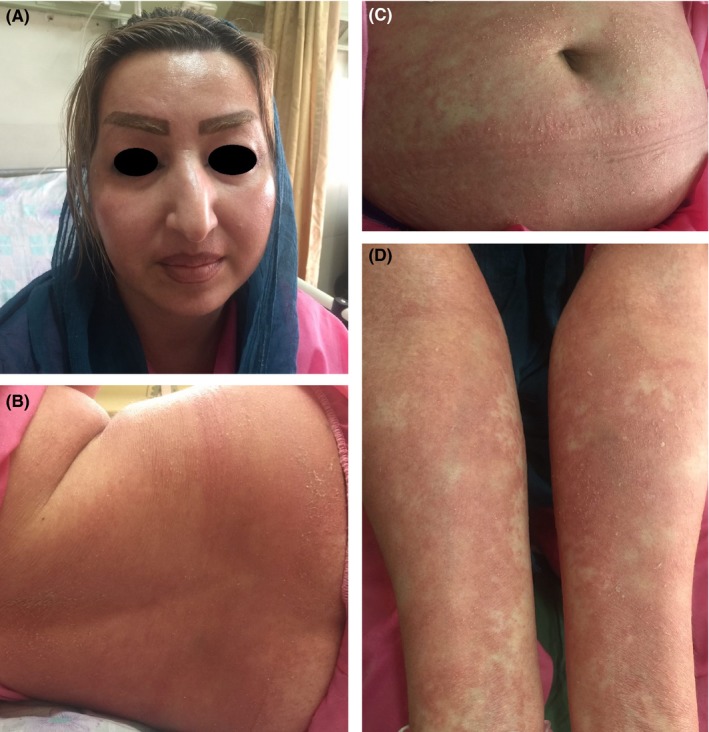
Facial involvement (A) small nonfollicular pustules on the back, abdomen and forearms on erythematous skin with typical extensive postpustular desquamation (B‐D)

She developed a wide pustular exanthema on her trunk and limbs that gradually spread on the face and scalp. Some annular erythematous lesions and erythematous patches with targetoid appearance with scale and studded nonfollicular pinpoint pustules were also seen on her legs (Figure 2).

Mucosal membrane, nail, and palmoplantar surface were spared.

She described the chills, lethargy, painful stinging, and pruritus sensation as the lesions spread. The patient had no personal/familial history of psoriasis.

She has a history of fever with a temperature of 38.7°C, but on admission, the vital signs were stable.

She had a high white blood cells count with a left shift (WBC 14 700, normal range, 4‐10 × 10^9^/L neutrophil count 10 900 equivalent to 86.1%).

Her septic workup including a CXR, blood culture, urine culture, and other routine laboratory including renal and liver function tests were unremarkable. Punch biopsy and swabs of the pustules were taken.

Bacterial and fungal cultures of the pustules were negative. The results of serologic screening for EBV, CMV, HBV, and mycoplasma were also negative. Later, we were informed that the initiation of hydroxychloroquine was due to a misdiagnosis and arthritis was ruled out by both laboratory and radiological investigations and her comfort was more related to arthralgia.

She was started topical steroids three times daily, antihistamine and oral prednisolone at a dosage of 35 mg daily. On second day, the eruption continued to persist and she reported development of new pustules and worsening of pain and pruritus. Antihistamine dosage was elevated and because of pathological demonstration of AGEP and rule out of psoriasis, prednisolone dosage was switched to 50 mg daily on fourth day.

After 15 days in hospital, the pustules and erythema were moderately improved and she had wide spread desquamations. She was discharged on tapering doses of oral prednisolone 50 mg/daily, antihistamine, and topical steroids twice daily. The patient visited 37 days after discharge with mild pruritic eruption and desquamation on upper limbs and trunk and distal lower extremities. She was instructed to taper prednisolone 5 mg weekly.

At a follow‐up 3 months later, the eruption was completely resolved with no recurrence and the systemic corticosteroid was tapered totally. She was also referred to a psychologist because of intermittent complaint about joint pain.

## 3 DISCUSSION

Acute generalized exanthematous pustulosis typically presents as a pustular rash with a diffuse often with acute onset, edematous that arises predominately intertriginous areas or on the face. Edema of the face/purpura, vesicle, blister, erythema multiform‐like lesions, and mucosal membrane involvement has also been described. Mild eosinophilia, transient renal failure, hypocalcemia, and elevated amino transferase levels may accompany fever and neutrophilic leukocytosis.^14^ Lymphadenopathy has described in some cases.^15^ Once the causative drugs have been discontinued, spontaneous resolution within 15 days occurs.^2^

Due to the benign self‐limiting course of AGEP, specific treatment usually is unnecessary in mild cases. Systemic corticosteroids are usually used for severe cases.^4^, ^5^, ^7^ Some authors have reported using dapsone and cyclosporine in treatment of severe and atypical forms of AGEP as case reports.^9^, ^13^

The pathogenesis of AGEP is not yet known although release of increased amount of IL_8_ by T cells with attraction and activation of polymorphous nuclear neutrophils has recently been proposed. And the disease is associated with human leukocyte antigens B51, DR11, and DQ3.^7^

Histologically, AGEP is composed by nonfollicular spongiotic pustules in the epidermis filled with neutrophils, papillary edema, and perivascular infiltration of neutrophils and associated eosinophilia.^3^

Differential diagnosis of AGEP includes bacterial folliculitis, varicella, impetiginized, eczema, staphylococcal‐scalded skin syndrome, and pustular psoriasis, that is, serologically, clinically, and histologically distinguishable. Distinguish between AGEP and generalized pustular psoriasis specially von Zumbusch‐type is important; recent drug administration, clinical course, and histopathological features help to distinguish between them. The pustules of pustular psoriasis are usually larger and show more prominent spongiform postulations, whereas necrotic keratinocytes, edema of papillary dermis, presence of eosinophils, mixed eosinophilic and neutrophilic infiltrations both in pustules and dermis, extravasation of erythrocytes, leukocytoclasia (rarely vasculitis), and absences of both tortuous blood vessels and significant epidermal psoriasiform changes are more prominent in AGEP.^3^, ^16^, ^17^

There are some reports of developing AGEP in patients with psoriasis, but no significant differences were observed between them and the patients with no history of psoriasis.^3^, ^7^

In AGEP, the average duration of drug exposure prior to onset of the symptom depends on the causative drug. Antibiotic consistently have been shown to trigger AGEP after 1 day, whereas often medication, including HCQ, averaged closer to 11 days.^18^

Hydroxychloroquine is an antimalarial medication with a half‐life of 1‐2 months, that is, a lysosomotrophic amin entering the lysosome of antigen‐presenting cells and raising the PH. HCQ is also an anti‐inflammatory agent and is thought to act by blocking the activities of toll‐like receptors on plasmocytoid dendritic cells. These features have to lead its use in the treatment of rheumatologic and dermatologic conditions.^19^, ^20^

Hydroxychloroquine has been described as a rare cause of AGEP. In a literature search by Paradisi and colleagues over 14 years between 1993 and 2007, only 16 report HCQ‐induced AGEP were reported.^7^

We also reviewed the literature in English since 2008 to 2018 and found nine other cases.^7^, ^8^, ^9^, ^10^, ^11^, ^12^, ^13^ The details of the cases are summarized in Table 1. The cases have been cleared within 8‐91 days. And our patient showed resolution after 68 days.

**Table 1:** Description of nine cases of hydroxychloroquine (HCQ)‐induced AGEP

	Age/sex	HCQ dosage	Time of onset	Treatment	Resolution of AGEP
Paradise et al^7^ (2008)	36/F	100 mg BID	21 d	Prednisolone 40 mg/d reduced By 10 mg every 4 d	8 d
	70/M	100 mg BD DC in 8 d	20 d	Prednisolone 40 mg/d	12 d
	79/M	100 mg BD D/C in 2 d	20 d	Prednisolone 40 mg/d	15 d
Lateef et al^8^ (2009)	67/F	Not reported	3 wk	Supportive for AGPE corticosteroid and IVIG for TEN	Hospitalized 2 W
Di Lernia et al^9^ (2009)	63/F	100 mg (BD) D/C 7 d Into rash	30 d	Cyclosporine	Exacerbation at 18 d, tapered corticosteroids
Park et al^10^ (2010)	38/F	200 mg D/C 7 d Into rash	3 wk	Methyl prednisolone 125 mg	Not reported
Bailey et al^11^ (2013)	48/F	200 mg HIQ	14 d	Methyl prednisolone prednisolone	18 d
Pearson et al^12^ (2016)	50/F	200 mg BD	2 wk	Methyl prednisolone 100 mg/daily	Exacerbation at 1th wk and 3th wk
Duman et al^13^ (2017)	42/F	200 mg daily	21 d	Methyl prednisolone 60 mg/daily Dapsone 50 mg/daily	35 d

These cases have history of initiation of HCQ within 2‐3 weeks prior to developing the symptoms, whereas in our patient, the eruption initially arose within 5 days.

## 4 CONCLUSION

Acute generalized exanthematous pustulosis is a drug‐induced pattern that often has a spontaneous resolution. HCQ is used extensively in rheumatologic and dermatologic conditions and is a rare cause of AGEP. Severe and prolonged cases of HCQ‐induced AGEP have been reported. Cessation is generally effective for therapy, but treatment is sometimes difficult. Thus, the physicians should take the possibility of this rare but severe event in their minds.

Recurrent and prolonged AGEP secondary to HCQ may be due to long half‐life of HCQ which is approximately 40‐50 days.

Utility of systemic corticosteroids in shortening the duration or decreasing the severity of the reaction has not been proven yet. Also efficacy of other drugs like dapsone or cyclosporine in this condition has not studied yet.

## CONFLICT OF INTEREST

None declared.

## AUTHOR CONTRIBUTION

MJ and FM: provided medical care, reviewed the medical records, interpreted data, and drafted the manuscript. FM and PR: provided pathological photographs and interpreted pathological data.
